# Materials Based on Co, Cu, and Cr as Activators of PMS for Degrading a Representative Antibiotic—The Strategy for Utilization in Water Treatment and Warnings on Metal Leaching

**DOI:** 10.3390/molecules28114536

**Published:** 2023-06-03

**Authors:** Efraím A. Serna-Galvis, Carlos Mendoza-Merlano, Ricardo A. Torres-Palma, Adriana Echavarría-Isaza, Dora A. Hoyos-Ayala

**Affiliations:** 1 Grupo de Investigación en Remediación Ambiental y Biocatálisis (GIRAB), Instituto de Química, Facultad de Ciencias Exactas y Naturales, Universidad de Antioquia UdeA, Medellín 050010, Colombia; ricardo.torres@udea.edu.co; 2 Grupo de Catalizadores y Adsorbentes (CATALAD), Instituto de Química, Facultad de Ciencias Exactas y Naturales, Universidad de Antioquia UdeA, Medellín 050010, Colombia; cjavier.mendoza@udea.edu.co (C.M.-M.); adriana.echavarria@udea.edu.co (A.E.-I.); 3 Grupo de Ingeniería y Gestión Ambiental (GIGA), Facultad de Ingeniería, Universidad de Antioquia UdeA, Medellín 050010, Colombia

**Keywords:** ciprofloxacin degradation, chromate of copper and cobalt, PMS activation, metal leaching, mixed metal oxides, water treatment

## Abstract

A chromate of copper and cobalt (Φy) was synthesized and characterized. Φy activated peroxymonosulfate (PMS) to degrade ciprofloxacin (CIP) in water. The Φy/PMS combination showed a high degrading capability toward CIP (~100% elimination in 15 min). However, Φy leached cobalt (1.6 mg L^−1^), limiting its use for water treatment. To avoid leaching, Φy was calcinated, forming a mixed metal oxide (MMO). In the combination of MMO/PMS, no metals leached, the CIP adsorption was low (<20%), and the action of SO_4_•^−^ dominated, leading to a synergistic effect on pollutant elimination (>95% after 15 min of treatment). MMO/PMS promoted the opening and oxidation of the piperazyl ring, plus the hydroxylation of the quinolone moiety on CIP, which potentially decreased the biological activity. After three reuse cycles, the MMO still presented with a high activation of PMS toward CIP degradation (90% in 15 min of action). Additionally, the CIP degradation by the MMO/PMS system in simulated hospital wastewater was close to that obtained in distilled water. This work provides relevant information on the stability of Co-, Cu-, and Cr-based materials under interaction with PMS and the strategies to obtain a proper catalyst to degrade CIP.

## 1. Introduction

Antibiotics are pharmaceuticals that are used worldwide to prevent and treat infections in humans and animals. The overuse of antibiotics in hospitals, agriculture, and livestock activities is promoting the development of antibiotic-resistant bacteria, which represents global environmental and health concerns [1,2]. Currently, effluents from hospital wastewater are considered a concerning source of releasing antibiotics into the environment [3,4]. Most of the antibiotics are recalcitrant to conventional water treatments [5]. Thus, the application of alternative processes able to eliminate antibiotics is urgently needed.

Advanced oxidation processes (AOPs) are alternatives that take advantage of short-lived and highly reactive oxygen species (ROS, e.g., HO•), which have been successfully utilized for the abatement of organic pollutants [6]. Typical AOPs, such as Fenton- and UV-based processes, generate ROS by activating hydrogen peroxide. Recently, AOPs utilizing peroxymonosulfate (PMS) or peroxydisulfate (PDS) (collectively referred to as persulfate-based AOPs) instead of hydrogen peroxide have emerged as an option to produce ROS and reactive sulfate species (RSS, e.g., SO_4_•^−^) to deal with organic pollutants in aqueous samples [7].

Among persulfate-based AOPs, the catalytic activation of PMS is a field of increasing interest in the scientific community because this kind of process allows the use of diverse homogeneous (such as Fe^2+^ or Co^2+^ in solution) or heterogeneous (such as transition metal oxides, activated carbons, biochars, or reduced graphene oxides) catalysts to easily promote the formation of ROS and RSS for degrading a wide variety of contaminants [7,8].

Cobalt is a key element in the heterogeneous catalytic activation of PMS. Cobalt-containing catalysts can be designed with a peculiar morphology or structure in order to enhance the efficiency of persulfate activation toward the production of ROS and RSS. In the heterogeneous catalytic activation of peroxides, it is also very common to utilize solids containing other transition metals, in addition to cobalt [9,10]. For instance, chromate species have been used to activate hydrogen peroxide for degrading organic pollutants in water [11]. Recently, chromium-substituted magnetite nanoparticles, or recycled chromium phosphate as a support of cobalt oxide, have been tested in the activation of PMS to deal with water contaminants [12,13]. However, to the best authors’ knowledge, still, the use of chromates of copper and cobalt and their corresponding oxides to activate PMS for the elimination of organic pollutants has not yet been reported.

In this work, chromate of cobalt and copper (Φy) and its corresponding mixed metal oxide (MMO, coming from Φy calcination) were prepared and used in a catalytic AOP. The catalysts’ stability (metals leaching and reuse) and the risk of releasing heavy metals from the catalysts into the solution were discussed. Both Φy and MMO were characterized and tested as activators of PMS for degrading a representative contaminant of emerging concern (i.e., ciprofloxacin, CIP). Φy was synthesized using a hydrothermal method. Once Φy was prepared and characterized, its activating ability was assessed, and the results suggested that the Φy/PMS system quickly degraded CIP. However, PMS promoted the dissolution of cobalt from the solid material, changing the system from a heterogeneous to a homogeneous catalysis. Considering the issues associated with metal leaching, Φy was calcinated to produce MMO. Then, the activation of PMS by MMO, activation pathways, and primary transformations were established. The change in the biological activity of CIP by the treatment was studied using theoretical analyses. Moreover, the treatment extent, testing the reuse cycles of the catalyst and the degradation of CIP in simulated hospital wastewater with the MMO/PMS system, was assessed. In the PMS/MMO process, the radical route dominated the target pollutant degradation and promoted the opening and the oxidation of the piperazyl ring and the hydroxylation of the quinolone moiety on CIP. The process presented high activation of PMS toward CIP degradation and the capability to change the biological activity of the antibiotic. After three reuse cycles, the MMO was still active toward PMS for the CIP degradation. Additionally, the CIP degradation in the simulated hospital wastewater was close to that obtained in distilled water.

## 2. Results and Discussion

### 2.1. Characterization of the Φy

**Figure 1:**
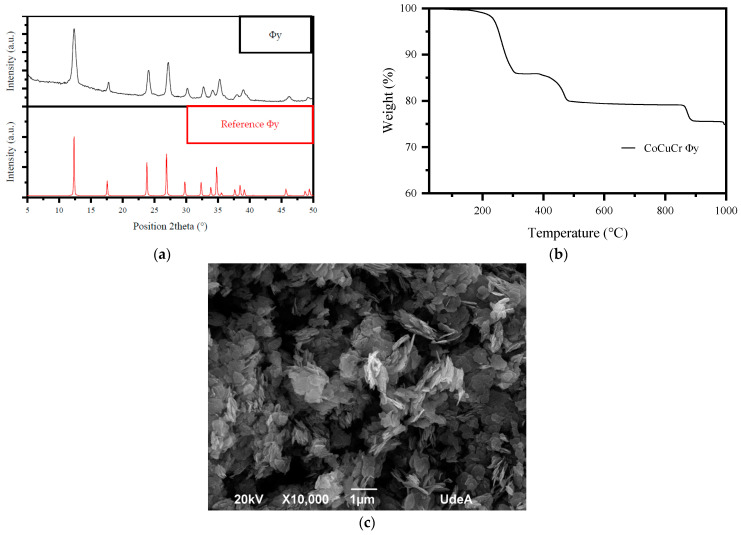
Characterization of chromate of copper and cobalt (Φy). (**a**) XRD pattern of Φy. (**b**) TGA for Φy. (**c**) SEM for Φy.

The chromate of copper and cobalt was synthesized by using the hydrothermal method and it was characterized using atomic absorption (composition), X-ray diffraction (XRD), Brunauer–Emmett–Teller analysis (BET), thermogravimetric analysis (TGA), and scanning electron microscopy (SEM). Figure 1a presents the XRD pattern of the synthesized material. This pattern shows a perfect correlation with the chromate phase Φy (corresponding to (NH_4_)_1.5_Co_x_Cu_2−x_Cr_2_O_8_ (OH)_1.5_·H_2_O, [14]) according to the ICSD database 98-009-3960. Table 1 summarizes the composition and the BET results. The content of Cu, Cr, and Co (which are relevant metals for the PMS activation) in Φy was at 28.3, 5.87, and 9.39% *w/w*, respectively. From the TGA analysis (Figure 1b), it was observed that, between 100 and 300 °C, there was an 18% loss of weight, due to the release of water and ammonium. The second event between 400 and 500 °C and the third event between 850 and 900 °C, having a 3.9% and 5.3% loss of weight, respectively, were due to oxygen desorption. In turn, the textural results presented in Table 1 indicate that half of the BET area mainly comes from the mesopore, which is associated with the external interparticle, and the rest of the area comes from the micropore interlaminar space. Additionally, the high total pore volume, calculated at p/p0 = 0.995, is mainly due to the high N_2_ uptake characteristic of nanometric-sized particles. In turn, Figure 1c (SEM micrograph) shows that the Φy material has particles with laminar morphology (flake-shaped particles), also supporting the textural analysis determined by N_2_ physisorption, with very well-defined crystals and with a lamellar extension that reaches up to microns.

### 2.2. Activation of PMS for the CIP Degradation

**Figure 2:**
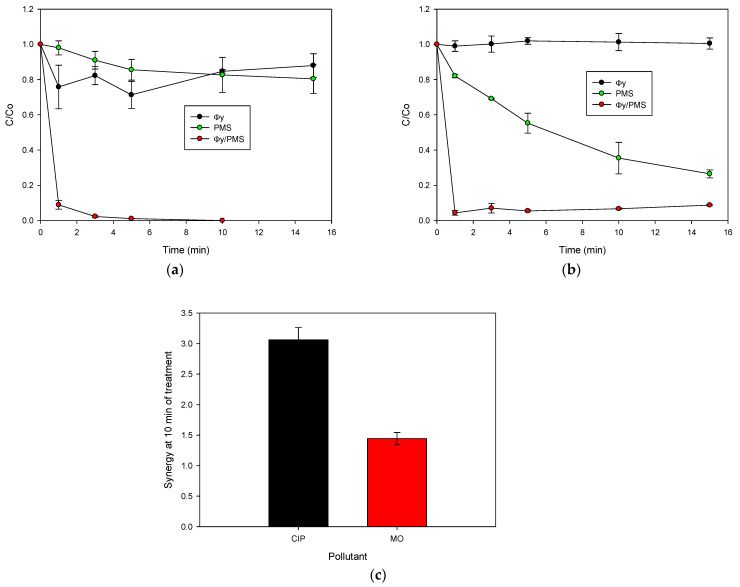
Treatment of organic pollutants with the Φy/PMS system. (**a**) CIP evolution, (**b**) MO evolution, and (**c**) synergy in the degradation of CIP and MO. Experimental conditions: CIP = 30.6 µmol L^−1^, MO = 30.6 µmol L^−1^, Φy = 0.2 g L^−1^, PMS = 500 µmol L^−1^, V = 50 mL, and initial pH = 5.4.

Initially, the adsorption of CIP on the Φy material and the direct oxidation by PMS were tested (Figure 2a). The Φy material alone showed a low adsorption capability toward the antibiotic (less than 20% of removal at 15 min), due to the low surface area (S_BET_: 38.2 m^2^ g^−1^) and the low pore volume (V_total_: 0.203 cm^3^ g^−1^) of this material (Table 1). In turn, the PMS degraded the probe pollutant to some extent (~15% after 15 min of interaction). This inorganic peroxide presents a high redox potential (E°: 1.82 V, [15]) and can directly oxidize CIP, thus leading to a decrease in the concentration of the antibiotic (Figure 2a). Interestingly, the combination of Φy with the PMS practically degraded the CIP after 3 min of treatment (Figure 2a), indicating the high activating capability of this material for degrading CIP.

After testing the ability of Φy to activate the PMS, the Φy/PMS combination was applied in order to treat another organic pollutant, testing the effect of the chemical structure of the contaminant on the process. Therefore, methyl orange (MO) was considered. The structural core of CIP is a quinolone system attached to a piperazyl ring, whereas MO has an azo group connecting two aromatic rings [16]. Figure 2b depicts the results of the treatment of MO. It can be noted that, after 10 min of treatment, the MO pollutant was not adsorbed by Φy. However, ~70% of the MO was degraded by direct oxidation of the PMS alone. Peroxymonosulfate is a strong oxidant that easily oxidizes MO, which has a lower redox potential (E: ~0.96 V [17]) than PMS. Despite the high degradation of the MO by the PMS alone, the Φy/PMS combination induced more than 90% of the pollutant removal after only 1 min of treatment, which highlights the high efficiency of the combination (Φy/PMS). 

For the degradation of both pollutants by Φy/PMS, the synergy was determined (calculated as % removal by the system/Σ and % removal by the individual components of the system) and compared (Figure 2c). The Φy/PMS system was highly synergistic. Indeed, the synergy values were 3.0 and 1.5 for CIP and MO, respectively, indicating that the enhancement of the degradation of the CIP by using the combined system (i.e., Φy/PMS), regarding the individual components (Φy or PMS), is higher than the improvement obtained for MO. Such enhancements can be associated with the generation and action of the degrading agents from the interaction of PMS with Φy (e.g., radical species) [7]. This aspect is developed in the following section.

### 2.3. Action Routes of the Φy/PMS System

**Figure 3:**
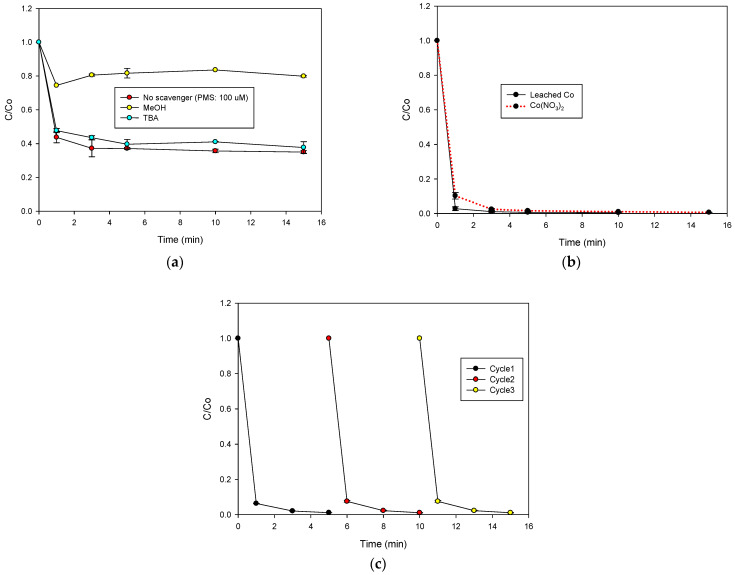
Elucidation of degradation routes and reuse cycles of the solid material in the Φy/PMS system. (**a**) Treatment of CIP in the presence of scavengers, (**b**) degradation of CIP by leached cobalt or cobalt nitrate, and (**c**) reuse of Φy in the Φy/PMS system. Experimental conditions: CIP = 30.6 µmol L^−1^, MeOH = TBA = 3060 µmol L^−1^, Φy = 0.2 g L^−1^, PMS = 500 µmol L^−1^ (for (**b**,**c**)), V = 50 mL, and initial pH = 5.6.

In order to establish the participation of radicals in the Φy/PMS process, the treatment of CIP was performed in the presence of methanol (MeOH, k_HO_^•^_/MeOH_: 9.7 × 10^8^ M^−1^ s^−1^, and k_SO4_^•−^_/MeOH_: 1.0 × 10^7^ M^−1^ s^−1^, [18]) or tert-butyl alcohol (TBA, k_HO_^•^_/TBA_: 6.0 × 10^8^ M^−1^ s^−1^, and k_SO4_^•−^_/TBA_: 8.4 × 10^5^ M^−1^ s^−1^, [18]). These scavengers were used 100-fold more concentrated than the pharmaceutical compound. Moreover, as the CIP degradation by the Φy/PMS process at 500 µmol L^−1^ of PMS is very fast (Figure 2a), the PMS amount was decreased up to 100 µmol L^−1^ in order to evidence the phenomenon. Figure 3a shows the evolution of the CIP in the absence and presence of the scavengers. MeOH inhibited the pollutant degradation, whereas TBA did not affect the CIP elimination. These results indicated that, in the Φy/PMS system, the peroxymonosulfate was activated toward the sulfate radical, which is the main species involved in the degradation of CIP. Furthermore, it should also be considered that the Φy material has copper and chromium elements in their highest oxidation states (2+ and 6+ for Cu and Cr, respectively, according to the XRD and TGA results), limiting the electron transfer (a typical mechanism involved in the activation of peroxymonosulfate [10]). In contrast, the cobalt element is in the oxidation state of 2+, and this metal could transfer an electron, which is very relevant for the activation of PMS [19,20,21].

As Φy is a solid material that contains Co, Cu, and Cr, an important aspect to be considered is the release of such metals from the solid into the solution [22]. Therefore, the contribution of homogeneous components in the Φy/PMS system by the leaching of metal ions was tested. Then, the interaction of the PMS with the solid material in the absence of pollutants was assessed, finding that ~1.6 mg L^−1^ of cobalt ions was released from the solid catalyst after 15 min of treatment. In addition, this lixiviate was used to treat CIP (Figure 3b), showing a very fast degradation of the antibiotic. Furthermore, a control experiment using homogeneous cobalt (i.e., Co^2+^ from Co(NO_3_)_2_ × 6H_2_O, at the same concentration lixiviated from Φy) was performed (Figure 3b), in which a very fast degradation of the target pollutant was also observed. All of these results evidenced that the homogeneous component has a very strong contribution to the degradation of the pollutants in the Φy/PMS system.

The addition of PMS to distilled water containing the Φy material decreased the pH from 5.4 to 3.4. In order to assess the role of a decreasing pH on cobalt ion leaching, other control tests were performed. An experiment evaluating the cobalt release when Φy is exposed to distilled water at pH 3.4 (adjusted using H_2_SO_4_) was carried out, indicating that 1.6 mg L^−1^ of cobalt ions were also leached under such conditions. Another control test, which consisted of the addition of Φy in distilled water containing PMS at pH 5.4 (previously adjusted using NaOH), showed the leaching of 0.9 mg L^−1^ of cobalt ions. Additionally, when Φy was added to distilled water at pH 2.4 (previously adjusted with H_2_SO_4_, with no presence of PMS), the solid was dissolved quickly and completely. It must be taken into account that, at an acidic pH, Co^2+^ predominates in a soluble form, and its redox potential increases according to the typical Pourbaix diagram for this metal [23], which can favor the activation of PMS, and, consequently, a fast elimination of CIP occurs. Therefore, all of the above results indicate that Φy is not stable during the pH decrease promoted by the PMS addition, thus leading to the release of cobalt ions (Equation (1)) and the promotion of the conversion of the initial heterogeneous system into a homogeneous process. Such a homogeneous system involves the interaction of the released cobalt to produce radical species (Equations (2) and (3), [24,25]), inducing a fast degradation of the target pollutants, as evidenced in Figure 2a,b.

Φy + HSO_5_^−^_(ac)_ → Co^2+^_(ac)_, (1)

Co^2+^_(ac)_ + HSO_5_^−^_(ac)_ → Co^3+^_(ac)_ + SO_4_•^−^ + HO^−^, (2)

Co^3+^ + HSO_5_^−^_(ac)_ → Co^2+^_(ac)_ + H^+^_(ac)_ + SO_5_•^−^, (3)

As the Φy material experiences cobalt leaching, it was very important to assess the activity when it was used for several cycles. Hence, the reusability of Φy was evaluated (Figure 3c). Additionally, the cobalt ions that were released after each cycle were also measured (Table S1, in the Supplementary Materials). It can be noted that, even after the third reuse cycle, Φy was able to endorse a fast elimination of CIP; however, the amount of leached cobalt ions decreased with each reuse cycle (Table S1). Such results indicate that, even though the leaching of cobalt was diminished after the cycles, the released ions activated PMS, producing enough radical amounts to completely degrade CIP.

It must be noted that, although cobalt ions are very efficient for the homogeneous activation of PMS, water containing such ions has a potential hazard to human health [10]. Even if the maximum permitted emission level for cobalt is low (<1.0 mg L^−1^), the long-term operation of leaching materials may induce the accumulation of toxic cobalt in water bodies. Furthermore, the secondary pollution caused by cobalt leaching from the materials during the catalytic activation of PMS should not be overlooked [22]. Indeed, cobalt leaching is a critical point (it is more worrying if cobalt was released at higher concentrations than the maximum permitted emission) that hinders the actual applications of the Φy material for water treatment. Therefore, as a strategy to inhibit cobalt leaching (which is also fairly beneficial for practical uses), the calcination of Φy was applied, generating a mixed metal oxide (MMO). Subsequently, the capability of the MMO to activate the PMS for CIP degradation was tested, as detailed in the following sections. 

### 2.4. PMS Activation Using MMO

**Figure 4:**
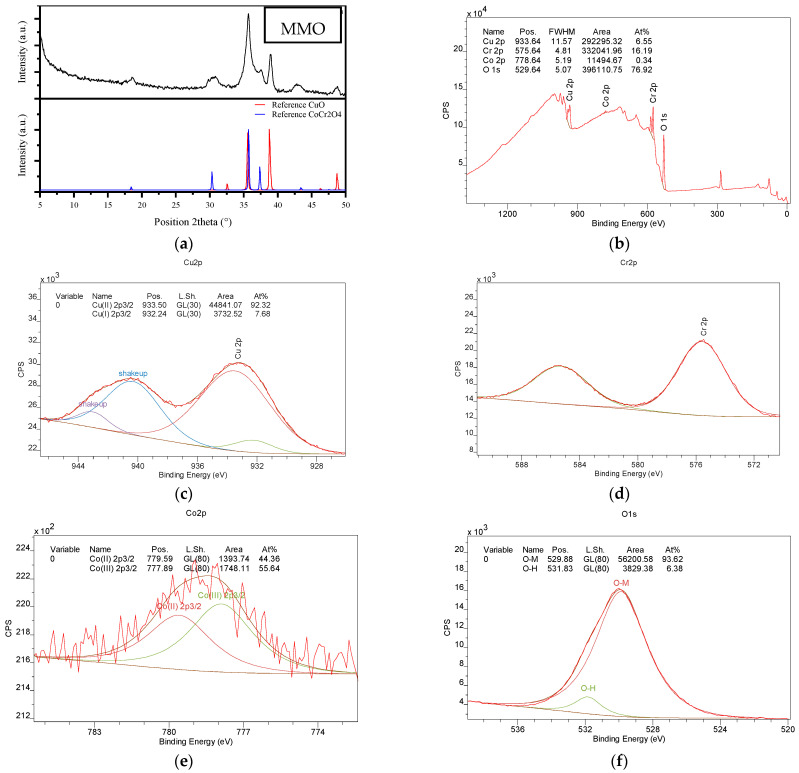
Characterization of MMO. (**a**) XRD, (**b**) XPS spectrum, (**c**) deconvolution of the peak for Cu, (**d**) deconvolution of the peak for Cr, (**e**) deconvolution of the peak for Co, and (**f**) deconvolution of the peak for O.

As mentioned above, the initial Φy was calcinated at 450 °C (such a calcination temperature was selected to produce the oxide based on the TGA analysis, Figure 1b), producing the MMO material [14]. XRD, BET, SEM, and composition analyses of the MMO were carried out (Figure 4 and Table 2). According to the XRD analyses for the calcinated material and its comparison with the patterns of ICSD 98-008-7126 and ICSD 98-002-7507) (Figure 4a), the MMO is composed of copper oxide (CuO) and cobalt–chromium oxide (Co_x_Cu_1_−_x_Cr_2_O_4_), having percentages of 28.5% and 71.5% (which were determined by the Rietveld method ([26], Figure S1), respectively. These aspects evidenced the destruction of the Φy framework and the generation of the mixed metal oxides of Co, Cr, and Cu, which is consistent with our previous work regarding the thermal decomposition of Φy-type materials [14].

XPS analyses for the MMO were also carried out (Figure 4b). The predominant presence of O and Cr, followed by Cu and Co, can be noted here. This figure also suggests that, in the MMO, the Co_x_Cu_1_−_x_Cr_2_O_4_ component is more exposed on the external surface, while CuO remains more internal. Moreover, from the deconvolution of the XPS peaks (Figure 4c–f), it was found that the surface of the MMO contained species of Co^2+^ (44.36%), Co^3+^ (55.64%), Cu^+^ (92.32%), Cu^2+^ (7.68%), Cr^3+^ (~100%), and O (93.62% belonging to the lattice). On the other hand, the N_2_ adsorption on the MMO (Figure S2a) showed an isotherm type IV, with a high N_2_ uptake at the high P/Po region, which is typical for materials with interparticle mesoporosity coming from the small particle size. In the absence of micropores, the external surface area provides the main contribution to the total pore volume, and the surface area corresponds to the mesopores among the particles (Table 2) [27]. From Figure S2b, which presents the SEM images for MMO, it can be noted that the calcination of the Φy induced a partial destruction of the flake-shaped particles (Figure 1c). However, the MMO particles inherited some morphological anisotropy from their parent Φy material. Moreover, the calcination process decreased the size of the particles below 1 µm, generating agglomerates of small particles with elongated shapes (Figure S2b).

**Figure 5:**
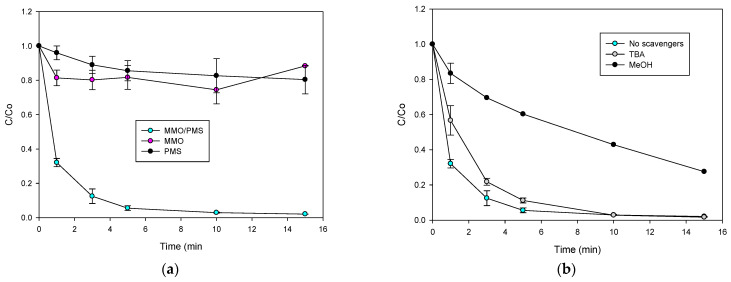
Treatment of CIP by the MMO/PMS combination. (**a**) Degradation of CIP and (**b**) treatment of CIP in the presence of radical scavengers. Experimental conditions: CIP = 30.6 µmol L^−1^, MeOH = TBA = 3060 µmol L^−1^ (only for (**b**)), MMO = 0.2 g L^−1^, PMS = 500 µmol L^−1^, V = 50 mL, and initial pH = 5.6.

After the characterization of the MMO, its capability for the adsorption and degradation of CIP was tested. Moreover, the leaching of the cobalt ions from the interaction of the MMO with the PMS was also evaluated. Figure 5a shows the adsorption of CIP on the MMO, the direct oxidation by the PMS, and the evolution under the MMO/PMS combination. A low adsorption of CIP on this material was observed (~20% after 15 min of treatment), which is explained by taking into account the low surface area (S_BET_: 36.8 m^2^ g^−1^) and pore volume (V_total_: 0.230 cm^3^ g^−1^) of the MMO (Table 2). In turn, the MMO/PMS combination led to a CIP elimination that was much higher than that obtained by adsorption or direct oxidation with PMS. Moreover, leaching of cobalt was not detected during the process, indicating a heterogeneous catalytic activation of PMS by the MMO. It is important to note that MMO has a degradation efficiency similar to that reported for cobalt-doped metal oxide/hydroxide (such as hierarchical Co(II)-doped TiO_2_, cobalt-doped biogenic manganese oxide, or Co-doped Fe_3_O_4_@FeOOH), substrates for heterogeneous PMS activation. However, those substrates have the disadvantage of the Co leaching [22], while MMO does not have this concern.

In order to establish the participation of radical species in the heterogeneous catalysis, experiments in the presence of MeOH or TBA were performed (Figure 5b). A strong inhibition of CIP degradation was found in the presence of methanol, while tert-butanol slightly decreased the pollutant removal. These results indicate a high participation of sulfate radicals and a low participation of hydroxyl radicals in the CIP elimination by the MMO/PMS system [18]. The generation of sulfate radicals (the main product responsible for CIP degradation) can be rationalized considering that the interaction of PMS with cobalt and copper ions on the MMO surface leads to sulfate radicals. Initially, =Co^2+^ and Cu^+^ can transfer an electron to PMS, forming SO_4_•^−^ (Equations (4) and (5)). The presence of Cu^+^ in the catalyst can also contribute to the inner reduction in =Co^3+^ because of the lower redox potential of =Cu^2+^/=Cu^+^ (E° = 0.16 V) than that of =Co^3+^/Co^2+^ (E° = 1.81 V) (Equation (6)) [22]; whereas, Cr_2_O_4_^2−^ acts as the carrier/support for the active metal for PMS decomposition. Furthermore, hydroxyl radicals can be formed through the interaction of sulfate radicals with water or hydroxyl anion in the liquid medium (Equations (7) and (8)) [24]. However, as also indicated by the results in Figure 5b, a small participation of HO• occurred.

=Co^2+^_(s)_ + HSO_5_^−^_(ad)_ → =Co^3+^_(s)_ + SO_4_•^−^ + HO^−^, (4)

=Cu^+^_(s)_ + HSO_5_^−^_(ad)_ → =Cu^2+^_(s)_ + SO_4_•^−^ + HO^−^, (5)

=Co^3+^_(s)_ + =Cu^+^_(s)_ → =Co^2+^_(s)_ + =Cu^2+^_(s)_, (6)

SO_4_•^−^ + H_2_O → SO_4_^2−^ + HO• + H^+^, (7)

SO_4_•^−^ + HO^−^ → SO_4_^2−^ + HO•, (8)

XRD, BET, and XPS analyses for the MMO after use in the activation of PMS were also performed (Figures S3–S5 and Table S2). These analyses showed that, under the experimental conditions, the structure, area, and composition of the surface of the used MMO were very similar to those of the MMO before use, indicating that this material effectively acted as a catalyst for PMS activation.

### 2.5. Primary Transformations of CIP Using the MMO/PMS System

**Figure 6:**
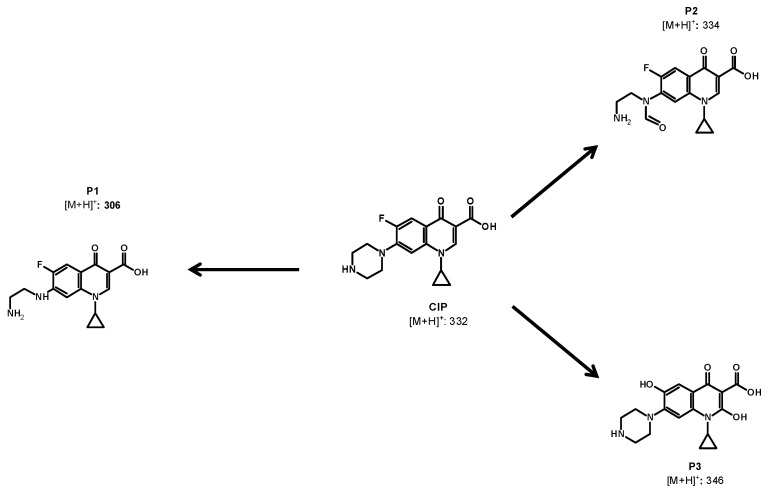
Primary transformation products from CIP degradation. Experimental conditions: CIP = 30.6 µmol L^−1^, MMO = 0.2 g L^−1^, PMS = 500 µmol L^−1^, V = 50 mL, and initial pH = 5.6.

In addition to the characterization of the used MMO, the action of radicals on the CIP structure was elucidated by determining the primary transformations by using LC–MS analyses (Figure 6). Three by-products of the CIP transformation were identified (P1, P2, and P3, for which MS spectra are presented in Figures S6–S9). The attacks of sulfate (or hydroxyl) radicals on the CIP structure led to the opening (P1) and the oxidation of the piperazyl ring (P2). Moreover, hydroxylations on the quinolone moiety induced the formation of P3. These transformation products are consistent with the computational calculations that have been reported in the literature [28], which indicate that piperazyl and the benzene rings are electron-rich moieties that are very susceptible to the reaction with radicals.

The formation of P1 and P2 can start with an electron transfer from the electron-rich piperazyl ring to the sulfate radicals [24,29,30,31]. The initial attack of SO_4_**•**^−^ at the secondary amine on the piperazyl moiety produces a cation radical, then, it experiences an α-deprotonation [32], with the posterior water reaction producing an intermediate alcohol. Such alcohol leads to imine and aldehyde groups. Subsequently, the hydrolysis of imine yields P2. In addition, the cation radical, which is initially generated from a radical attack to tertiary amine on the piperazyl ring, reacts with water, forming a secondary hydroxylamine. This hydroxylamine can evolve into a di-imine intermediate; moreover, afterward, the water can promote di-imine hydrolysis, yielding P1 [28,33]. Furthermore, the sulfate radical can also attack C-C π-systems [25]. As the quinolone system is an electron-rich region of CIP, an electron abstraction from such a moiety, followed by a reaction with water, generates the hydroxylation product (P3) [28,34].

The primary transformations induced by the MMO/PMS system could modify the antimicrobial activity (AA) associated with CIP. Therefore, in order to establish the possibility of the AA decreasing, theoretical analyses were carried out. Table 3 compares the probability (Pa) of the parent antibiotic and its primary products of being active. Biological activities such as the anti-infective, antibiotic quinolone-like DNA synthesis inhibitor, DNA gyrase inhibitor, DNA topoisomerase IV inhibitor, and topoisomerase II inhibitor (which are recognized as the main antimicrobial action mechanisms [35]) were considered.

It can be noted that all of the Pa values corresponding to the considered activities for the three primary products were lower than those for the CIP, suggesting a decrease in the AA after the treatment with the MMO/PMS system. This is also supported by the structural changes introduced to the parent antibiotic. The treatment modified the piperazyl ring and the quinolone group of CIP (Figure 6) that control the antibacterial potency, the action spectrum, and the efflux inhibition [36]. The cleavage of the piperazyl (P1) or oxidation (P2) may alter the acid/base speciation, decrease the lipophilicity, and diminish the cell permeability [37] and the linkage to bacterial DNA topoisomerases or DNA gyrase [38]. Moreover, the hydroxylation of the quinolone core (P3) and the loss of fluorine could also diminish the potency and the binding properties of the antibiotic [36]. 

**Table 3:** Predictions of the biological activity for CIP and its primary transformation products.

Biological Activity ^+^	Pa * for CIP	Pa for P1	Pa for P2	Pa for P3
Anti-infective	0.823	0.448	0.275	0.360
DNA synthesis inhibitor	0.786	0.529	0.493	0.500
Topoisomerase II inhibitor	0.751	0.581	0.505	0.313
Antibiotic Quinolone-like	0.567	0.172	0.138	0.053
DNA gyrase inhibitor	0.468	0.166	0.067	0.122
DNA topoisomerase IV inhibitor	0.222	0.070	0.052	0.031

**^+^** The predictions of biological activity were carried out on the PASS software (free online version, www.pharmaexpert.ru/passonline/index.php, accessed on 28 April 2023) [39]. * Pa: probability to be active.

### 2.6. Reuse of the Catalyst and Treatment in a Complex Matrix CIP Degradation and Using the MMO/PMS System

**Figure 7:**
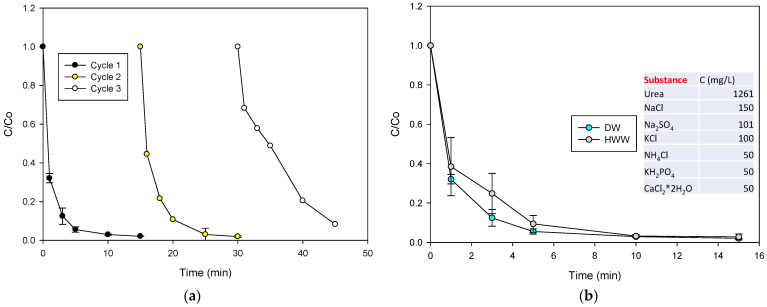
The extent of the treatment of CIP with the MMO/PMS system. (**a**) Reusability of the catalyst in the CIP degradation with the MMO/PMS system and (**b**) the degradation of CIP in HWW using the MMO/PMS system. Experimental conditions: CIP = 30.6 µmol L^−1^, MMO = 0.2 g L^−1^, PMS = 500 µmol L^−1^, V = 50 mL, and initial pH = 5.6.

The reusability of the catalyst and its performance in a matrix that is more complex than distilled water are influential factors for assessing the extent of the degrading processes [22,40]. Thus, the reuse test of the MMO catalyst in CIP degradation was considered. In addition, the treatment of CIP in synthetic hospital wastewater (HWW), using the MMO/PMS system was performed. Figure 7a presents the degradation of CIP after three reuse cycles of the catalyst. The CIP degradation rate decreased slightly after each cycle.

As occurs with other catalysts employed for PMS activation, the catalyst could adsorb some molecules of CIP, or its byproducts, onto the surface during the degradation process [41,42], and such adsorbed substances are not completely removed in the recycling tests. Therefore, a small portion of the active sites is occupied, resulting in a slight decrease in the catalytic performance of the MMO to activate the PMS and produce the radicals that are responsible for CIP degradation (see the S_BET_ results for the used MMO in Table 2, which had a slightly lower S_BET_ than the Φy material). However, it can be highlighted that this slight fall did not affect the application of the material significantly, because the MMO/PMS system was able to achieve up to 90% of pollutant removal, even after the third reuse cycle.

Regarding the treatment of CIP in the HWW (Figure 7b), it can be noted that the pollutant degradation was close to that obtained in the distilled water (DW). This could be explained by the fact that the MMO/PMS system can produce a lot of radicals to degrade the target molecule, with a low competence of the other matrix components. Moreover, the reaction of sulfate radicals with chloride anions is possible, which leads to the formation of chlorine radicals (Equation (9), [43]) that degrade CIP, even producing intermediates such as P1 (Figure 6) [44]. Additionally, PMS can also react with Cl^−^ to produce HOCl (Equation (10)) [45,46,47]. Hypochlorous acid is a well-known oxidizing agent capable of promoting CIP degradation [48,49]. Indeed, the interaction of CIP with HOCl also leads to the opening of the piperazyl ring (P1 in Figure 6) as the major transformation product [48].

SO_4_•^−^ + Cl^−^ → SO_4_^2−^ + Cl•, (9)

Cl^−^ + HSO_5_^−^ → HOCl + SO_4_^2−^, (10)

## 3. Materials and Methods

### 3.1. Reagents

Oxone^®^ (KHSO_5_, 0.5KHSO_4_, and 0.5K_2_SO_4_) was purchased from Sigma. The ciprofloxacin hydrochloride (CIP) was provided by Laproff Laboratories. The acetonitrile, sodium azide, sodium bicarbonate, sodium chloride, sodium dihydrogen phosphate, sodium hydroxide, sodium sulfate, sulfuric acid, methyl orange, and urea were provided by Merck. The ammonium chloride, calcium chloride dihydrate, magnesium chloride hexahydrate, and sodium citrate dihydrate were obtained from PanReac. The formic acid was obtained from Carlo Erba.

It is important to mention that CIP was selected as a representative pollutant because it is an antibiotic that is consumed worldwide, frequently found in diverse wastewater and environmental water [50]. CIP has particular concerns surrounding it due to its adverse effects on ecosystems. For example, CIP can promote the development and proliferation of antibiotic-resistant bacteria [51]. Additionally, this antibiotic belongs to the European watch list of substances for union-wide monitoring in water policy (Decision 2020/1161) [52].

The synthesis of the Φy material consisted of generating a laminar phase through the hydrothermal method, mixing the precursors of the metals required in the following specific molar ratio: CrO_3_:0.5Cu(NO_3_)_2_·3H_2_O:0.5Co(NO_3_)_2_·6H_2_O:NH_4_OH:164H_2_O. The solutions of CrO_3_ (Merck, Darmstadt, Germany, 99.0%), Cu(NO_3_)_2_.3H_2_O (Merck, 99.0%), and Co(NO_3_)_2_.6H_2_O (J.T Baker, 99.3%) were prepared in Teflon beakers. The pink cobalt solution was added in by stirring it into the blue copper solution, and, in the same way, the resulting mixture was added to the brown chromium solution. Afterward, the NH_4_OH (JT Baker, 28%) solution was added drop by drop, obtaining a brown gel that was stirred for 45 min and packed to a stainless-steel autoclave with a liner of Teflon. The reaction was carried out in an oven at 100 °C for 3 days. The obtained solid was filtered, washed to neutral pH, and dried at 60 °C. To obtain the mixed metal oxides of copper, cobalt, and chromium (i.e., MMO), the laminar phase (i.e., Φy) was calcinated at 450 °C for 1 h in an oven (Fisher Scientific, Pittsburgh, PA, USA) using an increase of 10 °C/min up to reach the calcination temperature.

### 3.2. Reaction Systems for Pollutant Removal

The catalytic tests were performed using a beaker in a magnetic stirring system (Figure S10, in the Supplementary Materials). A pollutant solution (50 mL) was considered, and the solid material (Φy or MMO) and PMS were added at 0.2 g L^−1^ and 500 µmol L^−1^, respectively. The concentrations of catalysts and peroxides were chosen based on previous works, which report the use of oxidants (e.g., PMS) in the range of 50–1000 µmol L^−1^ and 0.25–1.0 g L^−1^ for the solid catalysts as the suitable amounts for the activation process development [53,54,55,56,57,58].

### 3.3. Analyses

The X-ray diffraction analysis (XRD) was carried out in an XPert PANanalytical Empyrean series ii (Ni-filtered Cu Kα radiation), with a time-per-step of 50 s and a step size of 0.05° between 5 and 80° 2θ. The composition of the solid samples was analyzed in a Thermo Scientific Ice 3000 atomic absorption (AA) spectrometer. A total of 20 mg of sample was weighed and subject to acid digestion with 2.5 uL of concentrated HF at room temperature. The solutions were graduated to 100 mL and dilutions were made for the different metals. The thermograms were collected using a TA Instruments Discovery 650 analyzer and 25 mg of sample was weighed and heating to 800 °C, with a ramp of 10 °C min^−1^ and airflow of 40 mL min^−1^. An ASAP2020plus sortometer was used to obtain the nitrogen adsorption–desorption isotherms (NADI) after degassing 200 mg of sample at 250 °C for 4 h at 10 µm Hg. The specific surface area BET (S_BET_) was calculated by applying the Rouquerol method and a t plot was used for the external surface (S_ext_) area and micropore volume (V_micro_). The total pore volume (V_total_) was determined at 0.99 p/p_0_. The morphology of the materials was determined through scanning electron microscopy (SEM) using a JEOL JSM-6490LV (at 20 kV and 10,000× magnification).

The surface chemical information was determined by X-ray photoelectron spectroscopy (XPS) using a PHOIBOS 150 1D-DLD analyzer and monochromatic Al-K_α_ radiation (1487 eV) operated at 10 W. The XPS spectra were recorded with a pass energy of 83.36 eV and step size of 1 eV for general spectrums and a pass energy of 20 eV and step size of 0.1 eV for high-resolution spectra. The charge compensation was achieved by a low-energy electron flood gun (3 eV cathode voltage and 20 mA emission current). Adventitious C1s core level line was selected as the reference to calibrate the energy scale. The spectra were analyzed by using a Gaussian–Lorentzian blend (GL 20–30%) and a Shirley-type background subtraction.

The CIP evolution was followed using liquid chromatography. A Thermo Scientific Ultimate 3000 UHPLC, equipped with a DAD detector and an Acclaim^TM^ 120 Thermo Scientific column (C18, 5 µm, 4.6 × 150 mm), was utilized. In all cases, 20 μL was the injection volume. As the mobile phase, a 15/85 mixture of acetonitrile/formic acid (10 mmol L^−1^) at 1.0 mL min^−1^ was used. The detection wavelength was 278 nm. The primary transformation products from the CIP degradation were established using an HPLC Agilent 1200 series, coupled with an Agilent LC/MSD VL SQ mass spectrometer. The column and mobile phase operated at the conditions utilized for monitoring the pharmaceutical evolution. The injection volume was 10 µL, and the mass spectrometer detector was operated in positive ion mode [59]. In the case of MO, this was followed by measuring the absorbance at 465 nm using a UV5 Mettler-Toledo spectrophotometer.

For the target pollutant and its primary transformation products, predictions of their antimicrobial biological activity were carried out with PASS software (free online version) [39], which is based on analyses of structure–activity relationships [39]. The chemical structures of these substances were uploaded to the PASS software in the SMILE format individually (Table S3). Then, the PASS software output the values of the probability of biological activities (Pa) for the tested substances.

## 4. Conclusions

The chromate of copper and cobalt material (Φy) was effectively synthesized by the hydrothermal method. The Φy phase had a high crystalline nature, as shown by XRD and the laminar characteristics evidenced by its specific surface area. Additionally, the total pore volume demonstrated the small particle size, due to the space between the layers. This material showed a good ability to promote the degradation of CIP; however, the interaction with PMS destabilized this solid material, leaching metals and calling into question the practical application of Φy. Interestingly, the calcination of Φy produced an MMO, which had textural characteristics inherited from the laminar phase. Despite the elimination of microporosity, the MMO retained the interparticle mesoporosity. In addition, the phase of Co_x_Cu_1_−_x_Cr_2_O_4_ formed by calcination was favorably located on the surface of the material. Additionally, the MMO showed a degradation efficiency similar to that reported for cobalt-doped metal oxide/hydroxide substrates for heterogeneous PMS activation, with the advantage of no leaching of cobalt, which is a typical concern for the other substrates.

The MMO also presented a high activating capability toward PMS, stability, and no leaching of metals. In the MMO/PMS system, the generated sulfate radical species was responsible for the CIP degradation, as evidenced by the tests with scavengers (methanol and tert-butanol). The produced sulfate radical reacted with the target pollutant, opening the piperazyl ring of CIP and hydroxylating the quinolone moiety as the primary transformations (which decreased the biological activity according to the theoretical analyses). The MMO exhibited good reusability for the activation of PMS to degrade CIP, even after the third run. Furthermore, the MMO/PMS process showed a high degrading capability toward CIP in HWW, indicating that the complex matrix induced low competing effects. It can be remarked that this research has provided valuable information on the instability of Co- and Cu-based materials under interaction with PMS and a strategy to overcome the leaching limitation and obtain a proper catalyst (from Φy calcination) for promoting CIP transformation into more environmentally friendly compounds.

## Figures and Tables

**Table 1:** Elemental composition and BET results for Φy.

Sample	Metals * (%*w/w*)			
	Cu	Cr	Co	
Φy	28.30	5.87	9.39	
**Property**	**V_micro_ (cm^3^ g^−1^)**	**V_total_** **(cm^3^ g^−1^)**	**S_BET_** **(m^2^ g^−1^)**	**S_ext_** **(m^2^ g^−1^)**
Value	0.01	0.203	38.2	18.0

* Metals determined by AA, S_BET_: surface area BET, S_ext_: external surface area, V_micro_: volume of micropore, and V_total_: total volume.

**Table 2:** Main properties of the MMO.

Sample	Metals * (%*w/w*)			
	CuO		Co_x_Cu_1_−_x_Cr_2_O_4_	
MMO	28.5		71.5	
**Property**	**V_micro._ (cm^3^ g^−1^)**	**V_total_ (cm^3^ g^−1^)**	**S_BET_ (m^2^ g^−1^)**	**S_ext._ (m^2^ g^−1^)**
Value	0.00	0.230	36.8	36.8

* Metals determined by AA, S_BET_: surface area BET, S_ext_: external surface area, V_micro_: volume of micropore, and V_total_: total volume.

## Data Availability

Data will be available by request via email to the corresponding authors.

## Supplementary Materials

The following supporting information can be downloaded at: https://www.mdpi.com/article/10.3390/molecules28114536/s1, Figure S1: XRD Rietveld refinement for MMO; Figure S2: N_2_-adsorption isotherm and SEM for MMO; Figure S3: XRD analyses for the used MMO; Figure S4: N_2_-adsorption isotherm for the used MMO; Figure S5: XPS for the used MMO; Figures S6–S9: MS spectra; Figure S10: Reaction system; Table S1: Cobalt leaching from Φy; Table S2: Comparison of textural properties of the MMO before and after use; and Table S3: CIP and its degradation products in SMILES format.

## Sample Availability

Samples of the compounds are not available from the authors.
