# Supplementary material for: Materials Based on Co, Cu, and Cr as Activators of PMS for Degrading a Representative Antibiotic—The Strategy for Utilization in Water Treatment and Warnings on Metal Leaching
Source: Molecules. 2023 Jun 3;28(11):4536. doi: 10.3390/molecules28114536 (PMC10254359; doi:10.3390/molecules28114536)
Supplement: Supplementary file 1 [file molecules-28-04536-s001.zip › molecules-2400024-SI.pdf]

molecules

MDPI

SUPPLEMENTARY MATERIAL

Materials Based on Co, Cu, and Cr as Activators of PMS for Degrading a Representative Antibiotic—The Strategy for Utilization in Water Treatment and Warnings on Metal Leaching

Efraím A. Serna-Galvis [1,2,*], Carlos Mendoza-Merlano [2], Ricardo A. Torres-Palma [1], Adriana Echavarría-Isaza [2] and Dora A. Hoyos-Ayala [3,*]

[1] Grupo de Investigación en Remediación Ambiental y Biocatálisis (GIRAB), Instituto de Química, Facultad de Ciencias Exactas y Naturales, Universidad de Antioquia UdeA, Medellín 050010, Colombia; ricardo.torres@udea.edu.co
[2] Grupo de Catalizadores y Adsorbentes (CATALAD), Instituto de Química, Facultad de Ciencias Exactas y Naturales, Universidad de Antioquia UdeA, Medellín 050010, Colombia; cjavier.mendoza@udea.edu.co (C.M.-M.); adriana.echavarria@udea.edu.co (A.E.-I.)
[3] Grupo de Ingeniería y Gestión Ambiental (GIGA), Facultad de Ingeniería, Universidad de Antioquia UdeA, Medellín 050010, Colombia
* Correspondence: efrain.serna@udea.edu.co (E.A.S-G.); dora.hoyos@udea.edu.co (D.A.H-A.)

Table S1. Leaching of cobalt from Φy after each reuse cycle.

**Cycle**	**Leached Cobalt (mg L^{-1})**
1	1.6
2	1.3
3	1.1

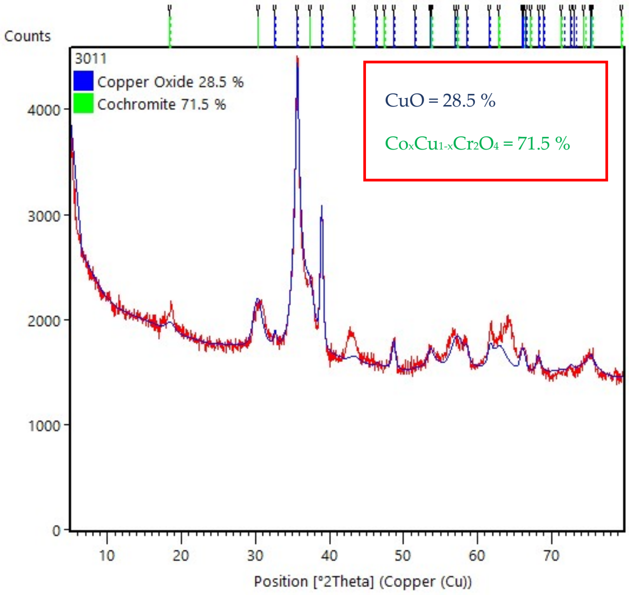

Figure S1. XRD Rietveld refinement for MMO.

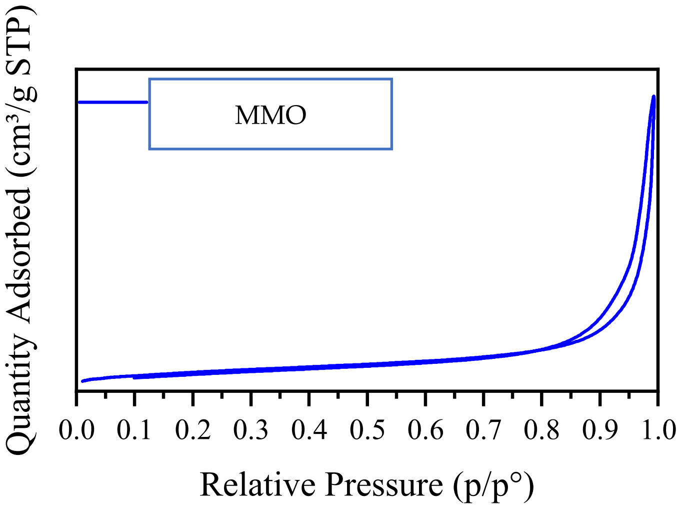

(**a**)

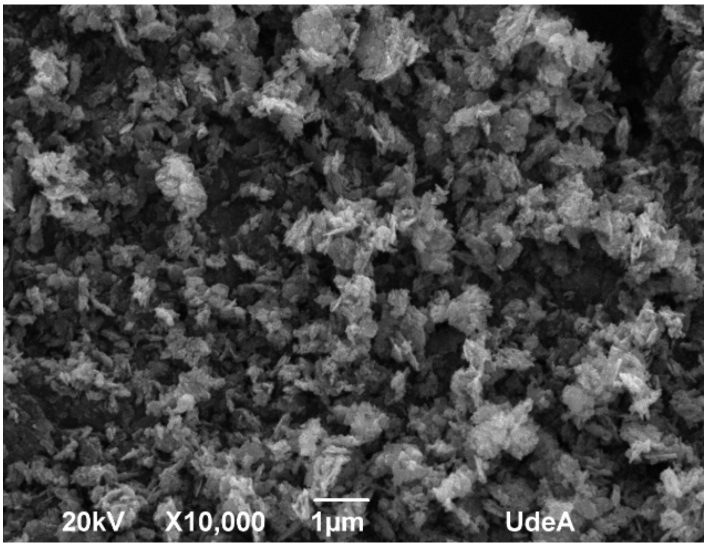

(**b**)

Figure S2. (**a**) The N_2-adsorption isotherm for MMO. (**b**) SEM for MMO.

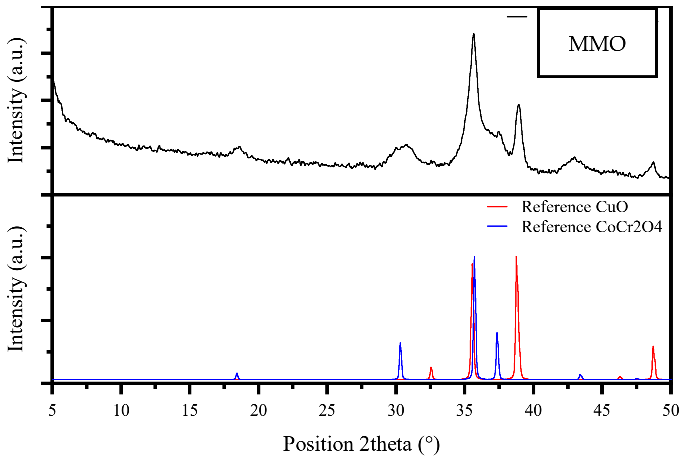

(**a**)

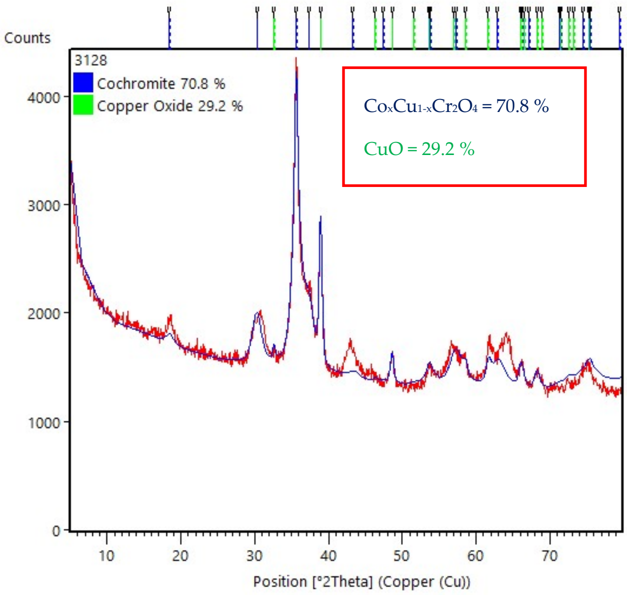

(**b**)

Figure S3. XRD analyses for MMO after its use in the MMO/PMS system. (**a**) XRD pattern and (**b**) Rietveld refinement.

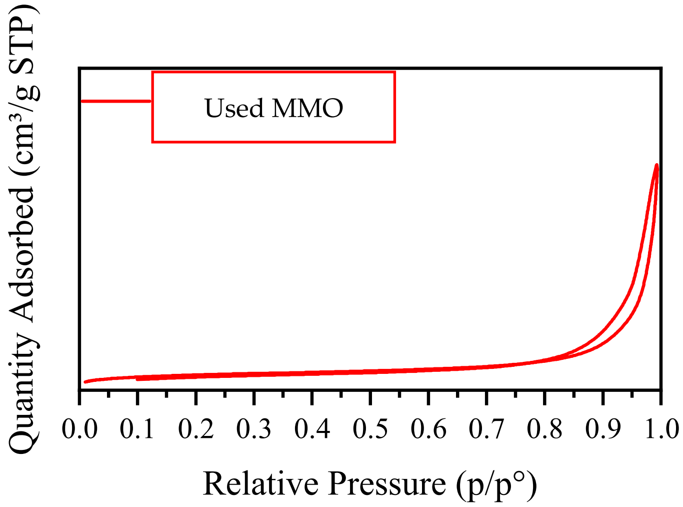

Figure S4. The N_2-adsorption isotherm for used MMO.

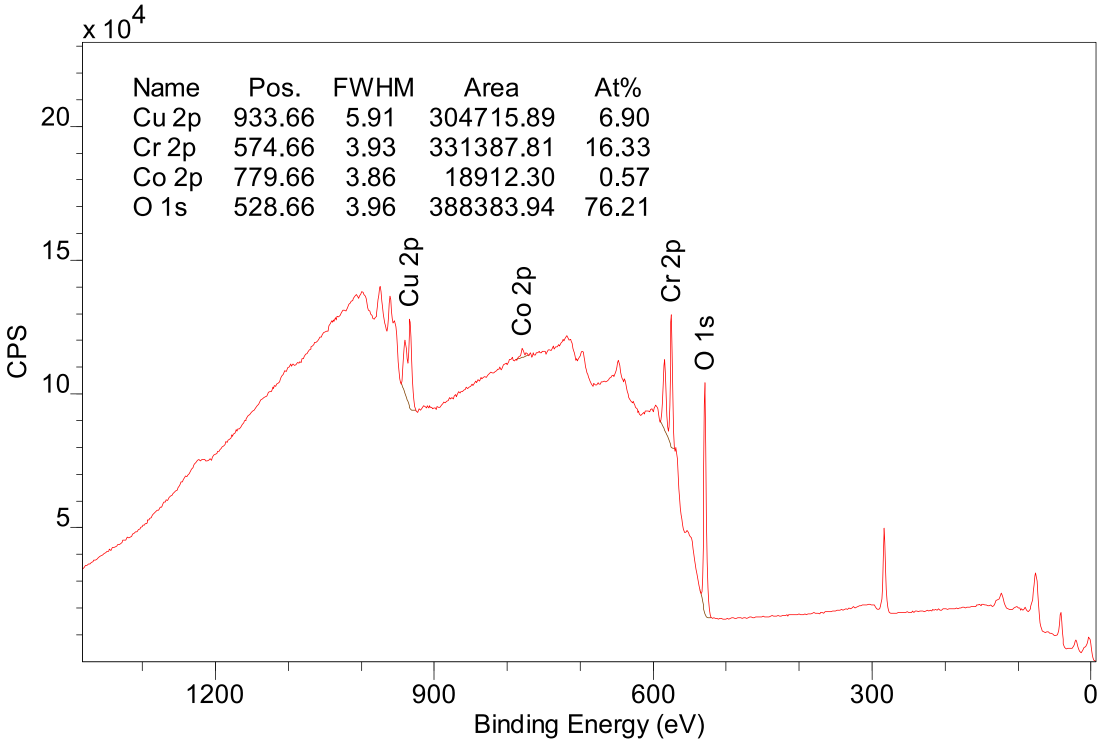

Figure S5. XPS for MMO after its use in the MMO/PMS system.

Table S2. Comparison of textural properties of MMO before and after use in the MMO/PMS system.

Material	$V_{micro.}$ ($cm^3\ g^{-1}$)	V_{total} ($cm^3\ g^{-1}$)	S_{BET} ($m^2\ g^{-1}$)	$S_{ext.}$ ($m^2\ g^{-1}$)
MMO before use	0.00	0.230	36.8	36.8
MMO after use	0.00	0.176	29.2	29.2

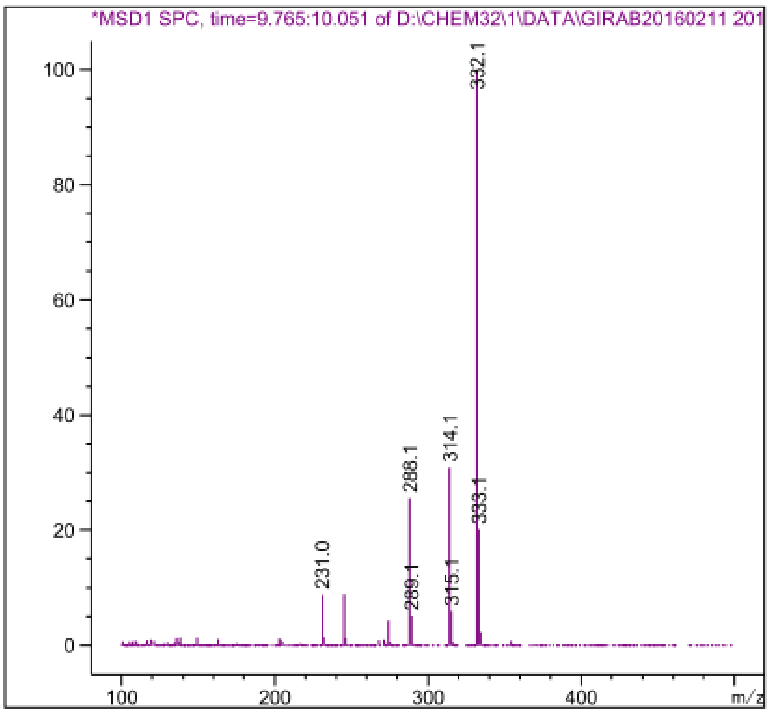

Figure S6. MS spectrum of CIP.

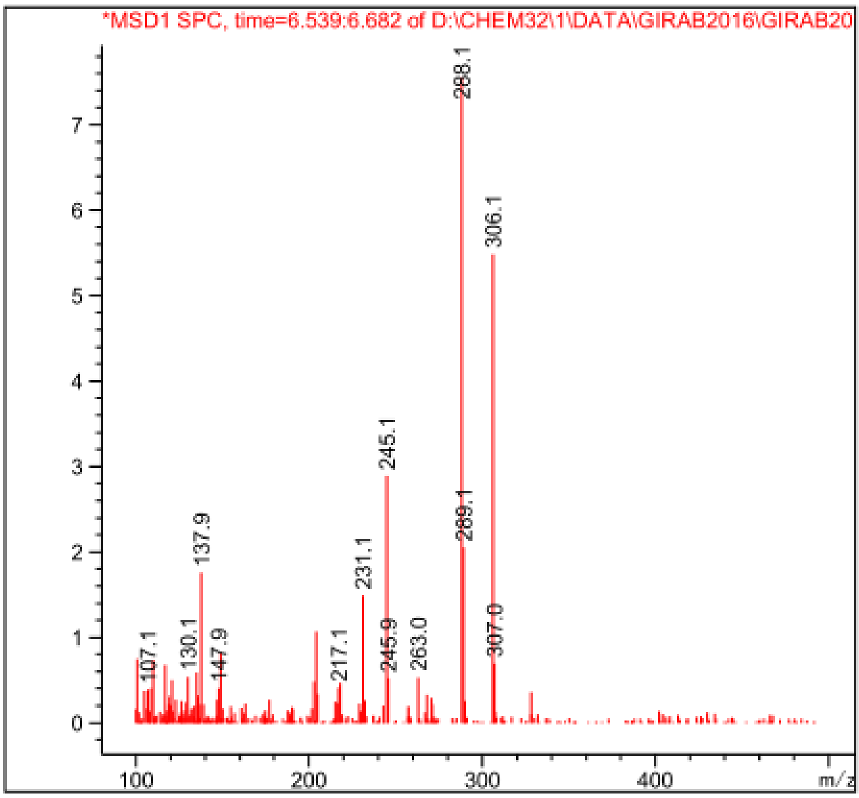

Figure S7. MS spectrum of P1.

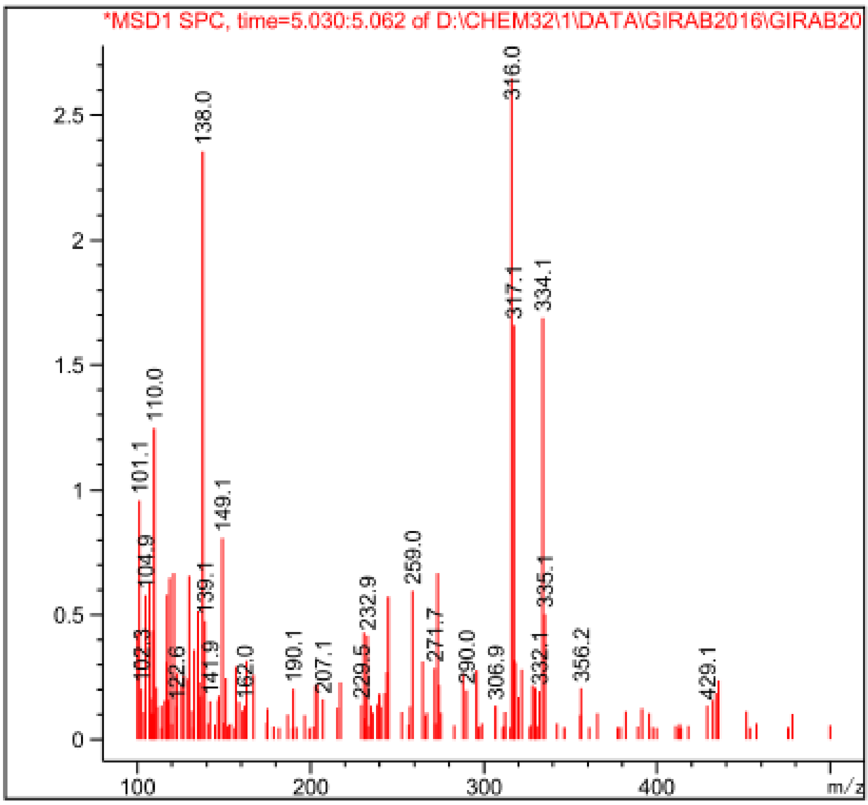

Figure S8. MS spectrum of P2.

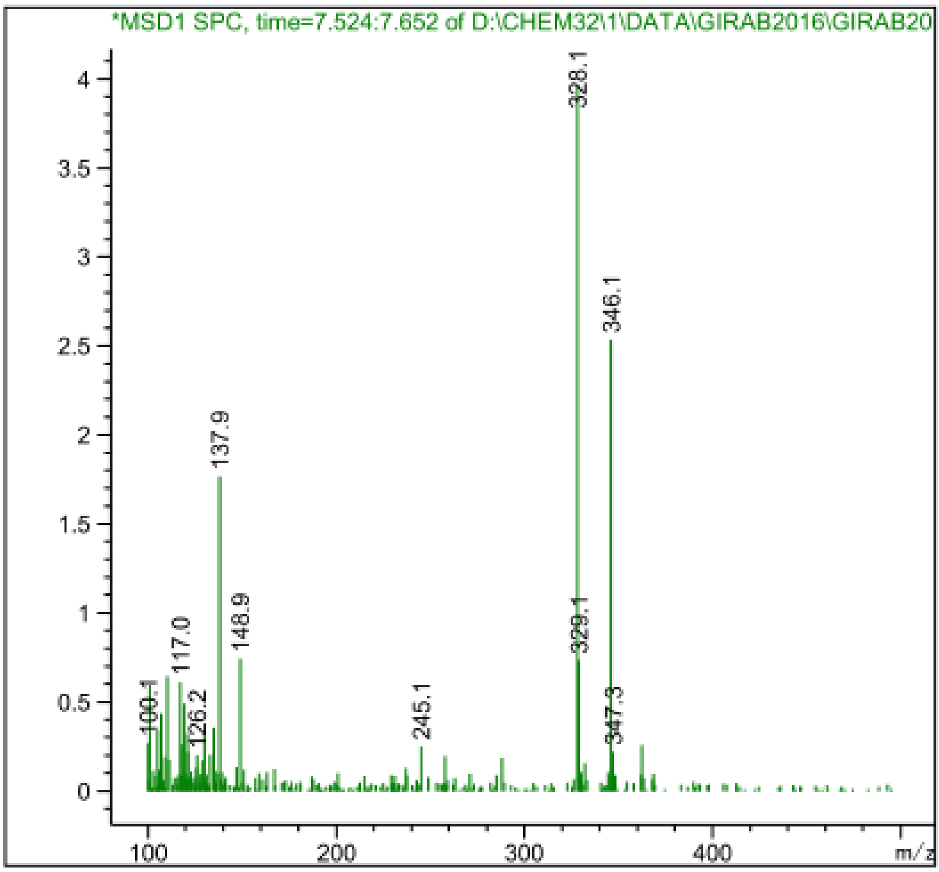

Figure S9. MS spectrum of P3.

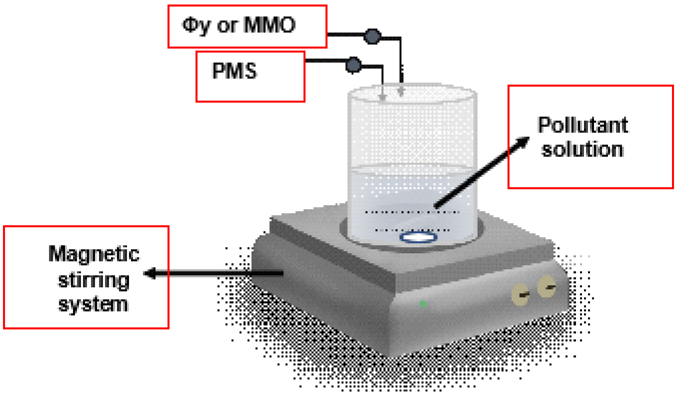

Figure S10. Reaction system for tests of pollutants degradation.

Table S3. CIP and its primary transformation products in SMILES format.

Substance	SMILES
CIP	O=C(O)C1=CN(c2cc(c(F)cc2C1=O)N1CCNCC1)C1CC1
P1	O=C(O)C1=CN(c2cc(NCCN)c(F)cc2C1=O)C1CC1
P2	O=CN(CCN)c1cc2c(cc1F)C(=O)C(=CN2C1CC1)C(=O)O
P3	O=C(O)C=1C(=O)c2cc(O)c(cc2N(C=1O)C1CC1)N1CCNCC1
